# HEALTH CARE ADMINISTRATION: HOW UPDATING OUR GERONTOLOGY UNDERGRADUATE PROGRAM INCREASED STUDENT ENROLLMENT

**DOI:** 10.1093/geroni/igae098.1617

**Published:** 2024-12-31

**Authors:** Nasreen Sadeq, Brian Kaskie, Debra Dobbs, Lindsay Peterson, Lu Norstrand, William Haley

**Affiliations:** University of South Florida, Tampa, Florida, United States; University of Iowa, Iowa City, Iowa, United States; University of South Florida, Tampa, Florida, United States; University of South Florida, Tampa, Florida, United States; University of South Florida, Tampa, Florida, United States; University of South Florida, Tampa, Florida, United States

## Abstract

The University of South Florida is a public research university in Tampa, FL with approximately 50,000 students across three campuses. The School of Aging Studies is one of several departments throughout the university that offers interdisciplinary undergraduate degree programs. However, like many gerontology programs across the country, the School of Aging Studies experienced a decline in undergraduate enrollment over the past decade and often struggled with generating enthusiasm for gerontology education and training. In order to continue our mission of providing an interdisciplinary gerontology education to undergraduate students, the School of Aging Studies performed an in-depth analysis of one of their undergraduate programs and determined that several updates were necessary to modernize the program and provide a more comprehensive education to students planning to work in the health care industry. In this presentation, we will describe our redeveloped Health Care Administration undergraduate program, including updates that were made to the curriculum and changes in program requirements. We will report on the intensive marketing and networking strategies that were developed with our university and community partners, and how our continued efforts led to the enrollment of approximately 40 students within one academic year. We will also describe our plans for the continued growth of the program and discuss the challenges that we have experienced and will continue to face in the future, including the increased demands placed on our faculty and staff, the availability of community internships available for our students, and the ability to sustain student interest in gerontology.

